# Professional language use by alumni of the Harvard Medical School Medical Language Program

**DOI:** 10.1186/s12909-020-02323-x

**Published:** 2020-11-06

**Authors:** Joseph A. Pereira, Kari Hannibal, Jasmine Stecker, Jennifer Kasper, Jeffrey N. Katz, Rose L. Molina

**Affiliations:** 1 Harvard Medical School, Boston, MA USA; 2 Department of Pediatrics and Global Health and Social Medicinem, Brigham and Women’s Hospital, Boston, MA USA; 3 Department of Medicine and Orthopedic Surgery, Brigham and Women’s Hospital, Boston, MA USA; 4 Department of Obstetrics and Gynecology, Beth Israel Deaconess Medical Center, 330 Brookline Ave, Boston, MA 02215 USA

**Keywords:** Medical language education, Medical Spanish, Medical Portuguese, Medical mandarin, Medical Haitian creole, Language-concordant care

## Abstract

**Background:**

Despite the growing number of patients with limited English proficiency in the United States, not all medical schools offer medical language courses to train future physicians in practicing language-concordant care. Little is known about the long-term use of non-English languages among physicians who took language courses in medical school. We conducted a cross-sectional study to characterize the professional language use of Harvard Medical School (HMS) alumni who took a medical language course at HMS and identify opportunities to improve the HMS Medical Language Program.

**Methods:**

Between October and November 2019, we sent an electronic survey to 803 HMS alumni who took a medical language course at HMS between 1991 and 2019 and collected responses. The survey had questions about the language courses and language use in the professional setting. We analyzed the data using descriptive statistics and McNemar’s test for comparing proportions with paired data. The study was determined not to constitute human subjects research.

**Results:**

The response rate was 26% (206/803). More than half of respondents (*n* = 118, 57%) cited their desire to use the language in their future careers as the motivation for taking the language courses. Twenty-eight (14%) respondents indicated a change from not proficient before taking the course to proficient at the time of survey whereas only one (0.5%) respondent changed from proficient to not proficient (McNemar’s *p*-value < 0.0001). Respondents (*n* = 113, 56%) reported that clinical electives abroad influenced their cultural understanding of the local in-country population and their language proficiency. Only 13% (*n* = 27) of respondents have worked in a setting that required formal assessments of non-English language proficiency.

**Conclusions:**

HMS alumni of the Medical Language Program reported improved language proficiency after the medical language courses’ conclusion, suggesting that the courses may catalyze long-term language learning. We found that a majority of respondents reported that the medical language courses influenced their desire to work with individuals who spoke the language of the courses they took. Medical language courses may equip physicians to practice language-concordant care in their careers.

## Background

Roughly 25 million individuals have limited English proficiency (LEP) in the United States (U.S.), with monolingual Spanish-speakers making up the largest share of this population [1]. Individuals with LEP seek health care services in nearly every U.S. health care system, highlighting the need for health professionals who are appropriately trained to address their needs linguistically and culturally [2]. As the number of patients with LEP grows, healthcare providers who can provide language-concordant care—defined as direct communication between patients and providers in the same language—are becoming more vital [3]. Language-discordant care, in which providers and patients are *not* proficient in a common language, leads to adverse health outcomes, such as physical harm, obstetric trauma, medication errors, communication barriers, minimal health education, and limited medical comprehension [4–9]. Conversely, language-concordant care has been associated with improved patient satisfaction, access and utilization of care, patient understanding of medical conditions, and objective clinical metrics (such as improved hemoglobin A1C levels) [10]. In addition, language-concordant care strengthens the therapeutic alliance between the physician and patient, as patients who perceive similarities with their providers are more likely to trust and adhere to recommended therapies [11, 12]. While qualified medical interpreters help to bridge communication gaps, their use has been hampered by cost, time, and lower patient satisfaction ratings when compared to encounters with language-concordant physicians [5, 13–15]. These challenges call attention to the importance of training physicians in language-concordant communication to provide the highest quality of care to a growing number of patients with LEP.

In order to address the current shortage of bilingual or multilingual physicians, many medical schools across the U.S. offer medical language courses [16]. In fact, a 2012–2014 survey found that 66% of U.S. medical schools offered a Medical Spanish curriculum, defined as a longitudinal program with multiple learning modalities [17]. However, there is no current estimate of how many schools offer non-Spanish medical language courses. Students who take Medical Spanish courses have shown improvements in listening comprehension skills, oral fluency, and comfort level working with Spanish-speaking patients [18–20]. In addition, a national survey found that 85% of medical students and trainees are interested in improving their Medical Spanish skills, suggesting that there is strong motivation to provide language-concordant care amongst trainees [21]. Yet, there is a dearth of information about the long-term impact of such medical language courses on physician use in professional settings, warranting further investigation. In addition, the literature lacks robust evaluation of non-Spanish medical language courses.

Since 2008, the Medical Language Program at Harvard Medical School (HMS) has offered intermediate level semester-long courses in Medical Mandarin, Portuguese, and Spanish in addition to advanced level Medical Spanish. Haitian Creole for health professionals, a beginner language course, was previously taught from 2005 to 2010. These semester-long courses include weekly evening sessions held for 1–2 h. Clinicians who are native speakers in the language teach the language courses; most had training in medical education and language instruction. The weekly classes are divided into three parts: grammar instruction, clinical vocabulary, and oral practice with native speakers. The Medical Language Program also offers an annual, month-long, intensive Medical Spanish course for both beginner and intermediate speakers with optional clinical electives in four Latin American countries. The month-long, intensive Medical Spanish course runs 6 h a day, 5 days a week (approximately 120 contact hours). Half of the day is committed to grammar instruction, taught by professionally trained linguists from a Latin American country. The other half of the day is dedicated to clinical vocabulary instruction from native Spanish-speaking physicians from Latin American countries and practical application, including oral practice with peers and daily hourly conversations with native-Spanish speakers from the community. Students who complete this course have the option to do a clinical rotation in one of several established clinical elective exchange programs in medical schools and hospitals in Latin American countries, typically held several months after the intensive course. Students who already demonstrate sufficient Spanish proficiency do not take the intensive Medical Spanish course but also have the option to participate in a clinical rotation at one of these sites. This rotation is conducted in conjunction with a partner medical school in the specialty of the students’ choice, pending availability. Between 1991 and 2007, the month-long, intensive Spanish course was the only credit-bearing medical language course offered at HMS. The HMS Medical Language Program has access to more than 800 alumni from the past several decades.

This study characterizes the professional language use of HMS alumni who took a medical language course at HMS and identifies opportunities to improve the HMS Medical Language Program.

## Methods

### Study design

This is a cross-sectional study, conducted between October and November 2019.

### Respondents

We included alumni who graduated from HMS between 1991 (the first year of available language course enrollment records) and 2019, who completed one or more HMS language courses, and who had an email address registered with the HMS Office of Alumni Affairs and Development. We excluded current HMS students, HMS graduates prior to 1991, and respondents who audited the courses or did not complete them. We included only graduates because we wanted to describe language use among alumni only. We obtained 804 email addresses for individuals who met inclusion criteria from the HMS Office of Alumni Affairs and Development. One individual replied indicating they were not the intended recipient of the survey, which resulted in a final total of 803 individuals surveyed.

### Assessment instruments

Our 36-item survey contains items about demographic information, linguistic background, language courses (including longitudinal semester-long language courses, intensive medical language courses, and clinical electives abroad), use of the language in a professional setting, and impact of the courses on respondents’ careers. We used the Interagency Language Roundtable Language Skill Level Descriptions as a standard scale for items about self-reported language proficiency [22]. Items about pre-course and post-course language skills were asked at the time of the survey completion based on current recollection. Items about course assessments refer to all language courses because some students took more than one course, with the exception of two items that asked specifically about clinical electives abroad (which appeared via branched-logic only if a respondent indicated that they took such electives). We created the survey with attention to best practices for medical education surveys, including conducting a literature review prior to item development, avoiding agree-disagree statement options, labelling all response options, and implementing pilot testing [23, 24]. We piloted the survey with three faculty and four current HMS students who had taken a medical language course to ensure clarity of questions and response options.

After incorporating feedback from the pilot testing, we administered the survey in October 2019, with three weekly email reminders sent to each participant. Participants who had already filled out the survey were asked not to fill it out again during the four-week period that the survey was available. We concluded collection of responses in early November 2019. Respondents who took language courses in multiple languages were asked only to answer questions about the language they use most in their professional careers. To reduce response bias, respondents answered the survey anonymously, and their responses were not linked to their email addresses. Any responses that were not marked as “completed” (*n* = 25), signifying that they began filling out the survey but did not submit their results, were removed from the final analysis. The complete survey is provided in the supplementary material.

We conducted qualitative coding of all open-ended responses using the consolidated criteria for reporting qualitative research [25]. Two primary coders used thematic analysis with formal coding to evaluate each response through an inductive approach. One of the coders was a medical student who previously took several medical language courses in the Medical Language Program, while the other was a research assistant with no prior exposure to the Medical Language Program. Any discordance in coding was settled by the principal investigator. Since no identifiers were collected, all investigators were blinded to the identity of the respondents. Specific themes were initially curated by consensus after the two primary coders analyzed all open-ended responses. Most responses did not exceed more than a few sentences, allowing us to limit the scope of our thematic analysis and increase inter-rater reliability. Coding was conducted in an Excel spreadsheet, with major themes given a binary ranking of “theme expressed” or “theme not expressed” for each response. For each question, the total number of occurrences for each theme was summed.

### Statistical methods

We summarized all survey responses using frequencies and percentages. We assessed changes in proportions over time using McNemar’s test to analyze paired data. For the questions assessing language proficiency, we categorized responses as “not proficient” (no practical, elementary, limited or minimum proficiency) or “proficient” (full or native/bilingual proficiency). For the question asking how frequently respondents expected to work with individuals that speak the language, we categorized responses as either “infrequently” (never, occasionally) or “frequently” (frequently, all the time) for the respective responses. We used JMP Pro version 14 for Mac (SAS Institute, Cary, North Carolina) for all data analyses. The institutional review board of the Harvard Faculty of Medicine determined that this study did not qualify as research.

## Results

We received completed responses from 206 of the 803 eligible alumni (response rate 26%), of which 137 (67%) identified as female and 190 (92%) were clinicians (Table 1). Nearly all of the respondents listed English as their primary or native language (*n* = 202, 98%); the most commonly identified race was white (*n* = 110, 53%), followed by Asian (*n* = 71, 34%), Black or African American (*n* = 19, 9%), and Hispanic, Latinx, or Spanish origin (*n* = 13, 6%). Of the eligible 803 alumni who took at least one language course, 588 (72%) took Intensive Medical Spanish, 147 (18%) took Intermediate or Advanced Medical Spanish, 36 (4%) took Medical Mandarin, 26 (3%) took Medical Haitian Creole, 15 (2%) took Medical Portuguese, and one (0.1%) was unknown. Ten individuals (1%) had taken more than one medical language course. The final sample of 206 respondents mirrored these results (Table 2). For example, 177 (86%) respondents took a Medical Spanish course, which is similar to the proportion of those sampled (*n* = 735, 90%). Of the eligible 803 alumni, 43 (5%) graduated between 1991 and 1995, 125 (16%) between 1996 and 2000, 129 (16%) between 2001 and 2005, 139 (17%) between 2006 and 2009, 78 (10%) between 2011 and 2015, 97 (12%) between 2016 and 2019, and 192 (24%) did not have a recorded graduation date on file with the Office of Alumni Affairs and Development (recorded as 2019 or earlier).

**Table 1:** Demographics of Alumni of Harvard Medical School’s Medical Language Program (*N* = 206, n (%))

Demographic Information	
*Age (years)*	
25–34	80 (39)
35–44	72 (35)
45–54	51 (25)
55–64	3 (1)
*Gender Identity*	
Female	137 (67)
Male	69 (33)
*Current Occupation*^a^	
Clinician	190 (92)
Research Investigator	51 (25)
Medical Educator	34 (17)
Other	10 (5)
*Self-Reported Racial/Ethnic Identities*^a^	
White	110 (53)
Asian	71 (34)
Black or African American	19 (9)
Hispanic, Latinx, or Spanish origin	13 (6)
Other^b^	11 (5)
*Primary or Native Language(s)*^a^	
English	202 (98)
Mandarin	18 (9)
Spanish	7 (3)
Polish	4 (2)
French	3 (1)
German	3 (1)
Portuguese	1 (0.5)
Other^c^	25 (12)

^a^Multiple responses were possible for these items

^b^The racial/ethnic groups “American Indian or Alaska Native,” “Arawak Indian of the Caribbean,” “Jewish,” “European,” “Middle Eastern,” “Pakistani/South Asian,” and “Persian” were each selected by one participant (0.5%). Two participants (1%) selected “Other” but did not disclose the group they identified most with. Two participants (1%) selected “Native Hawaiian or Other Pacific Islander.”

^c^Afrikaans, Hebrew, Arabic, Bengali, Cantonese, Czech, Hungarian, Korean, Teochew Chinese, Urdu and Yoruba were each selected as the primary/native languages of one participant (0.5%). Hindi, Russian, and Japanese were each selected by two (1%) participants each

**Table 2:** Language Courses Taken by Alumni of Harvard Medical School’s Medical Language Program (*N* = 206, n (%))

Medical Language Course Information				
*Language Course*				
Spanish			177 (86)	
Mandarin			18 (9)	
Portuguese			8 (4)	
Haitian Creole			3 (1)	
*Type of Language Course*^a^				
Intensive (month-long) language course			107 (52)	
Clinical elective abroad			94 (46)	
Longitudinal (semester-long) language course			66 (32)	
Unsure or other			9 (4)	
*Language Course by Self-Reported Racial/Ethnic Identities*				
Language Course	White (*N* = 110)	Hispanic, Latinx, or Spanish Origin (*N* = 13)	Black or African American (*N* = 19)	Asian (*N* = 71)
Spanish	105 (95)	11 (85)	18 (95)	53 (75)
Mandarin	1 (1)	0 (0)	0 (0)	17 (24)
Portuguese	3 (3)	2 (15)	1 (5)	0 (0)
Haitian Creole	1 (1)	0 (0)	0 (0)	1 (1)
*Pre-existing Language Experience Before Taking Medical Language Course*^a^				
Learned the language from formal classes in school				113 (55)
Beginner student or no prior experience				64 (31)
Lived/worked in a country where the target language was spoken				49 (24)
Learned the language from formal classes outside of school				37 (18)
Learned the language with family				24 (12)
Traveled to a country speaking the target language				2 (1)
Learned the language from friends				1 (0.5)

^a^Multiple responses were possible for these items

The majority of respondents took at least one course in Spanish (*n* = 177, 86%). Approximately half of respondents took a month-long language course (*n* = 107, 52%), while one-third took a semester-long course (*n* = 66, 32%). Medical Spanish had the highest enrollment across all four most commonly self-reported racial/ethnic identities (white: *n* = 105, 95%; Black or African American: *n* = 18, 95%; Hispanic/Latinx/Spanish origin: *n* = 11, 85%; Asian: *n* = 53, 75%). (Table 2).

The number (and percentage) of respondents whose self-reported proficiency improved from before the language course to the time of the survey is shown in Table 3. As noted in Methods, we created a binary proficiency indicator (with “proficient” defined as “full” or “native or bilingual” proficiency). Twenty-eight (14%) respondents indicated a change from not proficient before taking to the course (based on recollection) to proficient at the time of survey, whereas only one (0.5%) respondent changed from proficient to not proficient (McNemar’s *p* value < 0.0001). The frequency of higher-level proficiency (full and native/bilingual proficiency) increased among alumni of the three most common courses. The number (and percentage) of respondents whose expectation was to work with individuals who speak the target language increased between pre-course and post-course (based on recollection at time of the survey) is shown in Table 3. As indicated in the Methods, we created a binary expectation indicator (with “high expectation” defined as expecting to work with individuals who speak the target language “frequently” or “all the time”). Forty-five (22%) respondents indicated an increase to high expectation whereas only 12 (6%) respondents had decreased expectations (McNemar’s *p*-value < 0.0001). (Table 3).

**Table 3:** Self-Reported Oral Language Proficiency and Expectation to Work with Individuals Speaking the Target Language Among Alumni of Harvard Medical School’s Medical Language Program (*N* = 206, n (%))

Self-reported oral language proficiency						
	*Before Taking the Language Course*		*Current Language Proficiency*		*P*-value^a^	
No practical proficiency	51 (25)		3 (1)		*p* < 0.0001	
Elementary proficiency	54 (26)		31 (15)			
Limited working proficiency	62 (30)		80 (39)			
Minimum proficiency	27 (13)		53 (26)			
Full proficiency	6 (3)		32 (16)			
Native or bilingual proficiency	6 (3)		7 (3)			
**Change in self-reported oral language proficiency**^b^						
Number of respondents who changed from *not proficient* to *proficient*			28 (14)			
Number of respondents who changed from *proficient* to *not proficient*			1 (0.5)			
**Self-reported oral language proficiency stratified by course**						
	Longitudinal (semester-long) language course (*N* = 66, n (%))		Intensive (month-long) language course (*N* = 107, n (%))		Clinical elective abroad (*N* = 94, n (%))	
	*Before*	*Current*	*Before*	*Current*	*Before*	*Current*
No practical proficiency	8 (12)	1 (2)	39 (36)	2 (2)	20 (21)	0 (0)
Elementary proficiency	14 (21)	11 (17)	33 (31)	18 (17)	22 (23)	3 (3)
Limited working proficiency	26 (39)	20 (30)	29 (27)	51 (48)	34 (36)	39 (41)
Minimum proficiency	15 (23)	22 (33)	5 (5)	22 (21)	9 (10)	27 (29)
Full proficiency	2 (3)	12 (18)	1 (1)	13 (12)	4 (4)	18 (19)
Native or bilingual proficiency	1 (2)	0 (0)	0 (0)	1 (1)	5 (5)	7 (7)
**Expectation to work with patients or other colleagues who speak the target language**						
	*Before the course(s) began*		*After course completion*		*P*-value^a^	
Never	11 (5)		1 (0.5)		*p* < 0.0001	
Occasionally	106 (51)		83 (40)			
Frequently	77 (37)		105 (51)			
All the time	12 (6)		17 (8)			
**Change in expectation to work with patients or other colleagues who speak the target language**^c^						
Number of respondents who changed from *low or no expectation to high expectation*					45 (22)	
Number of respondents who changed from *high expectation* to *low or no expectation*					12 (6)	

^a^McNemar’s test for comparing proportions with paired data

^b^Proficient defined as “full” or “native or bilingual” proficiency

^c^High expectation defined as “frequently” or “all the time”

At the time of the survey, more than half of respondents reported spending less than 1 h per week speaking the target language (*n* = 134, 65%) (Table 4). Most had at least some interaction with someone speaking the target language (*n* = 152, 74%) or worked in a setting where at least some patients speak the target language (*n* = 156, 96%). The majority of individuals spoken to in the target language in a professional setting were patients (*n* = 144, 70%). Very few respondents (*n* = 27, 13%) took a formal language proficiency assessment in their professional setting, but of those who did, 20 (74%) reported the formal assessment was required.

**Table 4:** Use of non-English Languages in Current Professional Setting Among Alumni of Harvard Medical School’s Medical Language Program (*N* = 206, n (%))

Medical Language Use in Current Professional Setting	
*Hours Currently Spent Speaking Target Language Per Week*	
0	44 (21)
> 0 but < 1	90 (44)
1–5	49 (24)
6–10	13 (6)
11–20	6 (3)
21–40	2 (1)
41+	2 (1)
*Number of Individuals Spoken to in Target Language Per Week*	
0	54 (26)
1–5	112 (54)
6–20	32 (16)
20+	8 (4)
*Estimated Proportion of Patient Population that Speaks Target Language, N* = 163^b^	
0	7 (4)
> 0 but ≤10	80 (49)
11–25	36 (22)
26–50	30 (18)
51–75	7 (4)
75–100	3 (2)
*Individuals Typically Spoken to in the Target Language in a Professional Setting*^a^	
Patients	144 (70)
Patient’s family members	119 (58)
Colleagues or employees	21 (10)
Community Members	1 (0.5)
*Formal Language Proficiency Assessment in Professional Setting*	
Formal assessment taken	27 (13)
Assessment required	20 (74)

^a^Multiple responses were possible for these items

^b^The N is less than 206 (total respondents) because this question was open-ended and not all respondents responded

More than half of respondents (*n* = 118, 57%) were substantially motivated to take the medical language course (including longitudinal semester-long language courses, intensive medical language courses, and clinical electives abroad) because they wanted to use the language in their future careers, whereas only a quarter (*n* = 24, 12%) were substantially motivated by family or heritage influence (Table 5). Two-hundred respondents (97%) reported that the medical language courses were helpful in improving their oral proficiency of the target language in their careers. The courses similarly helped respondents understand the social determinants of health of those speaking the language (*n* = 192, 93%). The majority of respondents (*n* = 160, 78%) reported the courses have influenced their careers. Specifically, of the 113 (55%) who participated in a clinical elective abroad, all stated the clinical elective influenced both their cultural understanding of the local population abroad and their language proficiency.

**Table 5:** Motivation for Taking the Language Course and Influence on Career Trajectory

Motivation for Taking the Language Course(s) ***N*** = 206, n (%)	
*Desire to use the language in future career*	
Not at all motivated	4 (2)
Slightly motivated	7 (3)
Moderately motivated	29 (14)
Motivated	48 (23)
Substantially motivated	118 (57)
*Desire to use the language in personal life apart from future career*	
Not at all motivated	15 (7)
Slightly motivated	33 (16)
Moderately motivated	50 (24)
Motivated	47 (23)
Substantially motivated	61 (30)
*Desire to learn more about social determinants of health of those who speak the language*	
Not at all motivated	13 (6)
Slightly motivated	33 (16)
Moderately motivated	52 (25)
Motivated	52 (25)
Substantially motivated	56 (27)
*Family/heritage influence*	
Not at all motivated	108 (52)
Slightly motivated	29 (14)
Moderately motivated	31 (15)
Motivated	14 (7)
Substantially motivated	24 (12)
**Helpfulness of the Language Course(s)** ***N*** **= 206, n (%)**	
*Oral proficiency of the target language in participants’ medical career*	
Not at all helpful	6 (3)
Slightly helpful	19 (9)
Moderately helpful	46 (22)
Helpful	59 (29)
Substantially helpful	76 (37)
*Oral proficiency of the target language in settings outside of medicine*	
Not at all helpful	13 (6)
Slightly helpful	42 (20)
Moderately helpful	52 (25)
Helpful	55 (27)
Substantially helpful	44 (21)
*Understanding the social determinants of health of those speaking the target language, including patients’ social and cultural contexts*	
Not at all helpful	14 (7)
Slightly helpful	25 (12)
Moderately helpful	59 (29)
Helpful	59 (29)
Substantially helpful	49 (24)
**Influence of Medical Language Courses on Career** ***N*** **= 206, n (%)**	
Not at all influential	46 (22)
Slightly influential	32 (16)
Moderately influential	49 (24)
Influential	47 (23)
Substantially influential	32 (16)
**Influence of Clinical Elective Abroad** ***N*** **= 113, n (%)**	
*Cultural Understanding of the Local In-Country Population*	
Not at all improved	0 (0)
Slightly improved	1 (1)
Moderately improved	3 (3)
Improved	32 (28)
Substantially improved	77 (68)
*Oral Language Proficiency*	
Not at all improved	0 (0)
Slightly improved	5 (4)
Moderately improved	15 (13)
Improved	27 (24)
Substantially improved	66 (58)

In the open-ended responses, respondents most commonly reported that the language course(s) influenced their careers by enabling them to communicate with patients or patients’ family members. Respondents listed suggestions to improve the Medical Language Program, including more intensive and more longitudinal courses. Furthermore, many respondents suggested that the courses could be improved by offering more practical experience. Respondents reported that immersion courses were the best method of learning about socio-cultural contexts, but a few also commented that the courses did not address cultural issues. (Supplementary Table 1).

## Discussion

Our study reveals important information regarding the HMS Medical Language Program, which will help inform improvements in our own curricula and may assist other medical language programs at other institutions. Our findings have implications for clinical practice and communication with patients with LEP.

We found that alumni were motivated to take medical language courses because of their desire to use the language in their careers, with most taking courses in Medical Spanish. This suggests that the alumni took the courses to help facilitate care for Spanish-speaking individuals, who make up the largest population of patients with LEP in the U.S. [1] As such, medical school educators developing their own medical language courses should consider the common non-English languages spoken in their surrounding population. While our findings add to the literature by including non-Spanish medical language courses, we are limited by a small number of responses from those who enrolled in those courses.

Alumni reported improved oral proficiency over time. Many factors may contribute to this improvement, such as frequency of language use in personal and professional settings, travel to countries where the language is spoken, and additional courses taken since graduating from HMS. Nevertheless, our findings suggest that in general, the self-reported langauge proficiency of alumni improved since taking the course, supporting other reports that have shown improvement in the oral proficiency of students before and after taking medical language courses [18–20]. Acknowledging that it has been several decades since some respondents took a medical language course at HMS, we cannot conclude that the courses alone are responsible for the increase in proficiency. The open-ended responses offer an explanation for language proficiency improvement over time, as respondents commonly reported that the courses catalyzed life-long medical language learning and acquisition. In addition, based on current linguistic theory, teaching a medical language in a clinical context can allow for cue-dependent language retrieval when in a professional setting, mitigating language attrition [26]. It would be particularly helpful to assess proficiency longitudinally, following students’ language skills before taking a course, after course completion, and at timepoints post-graduation, but the feasibility of conducting such a prospective study remains challenging. Our findings add to the current literature about how medical school language courses affect physicians’ careers.

Our findings reveal that formal competency assessments were not commonly used to evaluate the respondents’ language proficiency in a professional setting. This is lower than reported in a 2009 nationally representative survey of U.S. hospitals, which found that 18% of hospitals require such assessments [27]. Without formal language proficiency assessments, providers who have only limited proficiency in a language may inappropriately rely on their own skills rather than utilize interpreter services [15]. Inadequate language proficiency can lead to inadvertent mistakes and worse outcomes for patients with LEP [5–7]. Furthermore, many U.S. medical schools do not consistently link their medical language courses to competency assessments [28]. Not all students taking language courses achieve a proficiency level that would enable them to provide safe care for patients with LEP. One study found that a majority of trainees with less than conversational Spanish language skills reported taking a history or providing medical advice without adequate interpretation services [21]. As such, formal assessments should be included upon course completion to benchmark progress toward professional proficiency. We propose that offering formal language proficiency assessments will help students determine appropriate use of their language skills in clinical encounters. As Ortega and colleagues have recently cited, the current lack of robust medical language assessment may lead to inappropriate use of presumptive language skills [28].

We found that a majority of respondents reported that the medical language courses influenced their desire to work with individuals who speak the language of the courses they took. This suggests that the courses may motivate students to work with linguistically diverse populations in their careers. The themes reflected in the open-ended responses also support this finding, with alumni frequently reporting that the course influenced their choice of clinical setting or patient population. These programs appear to not only attract students who are already more likely to care for such patients, but also increase the likelihood that they will do so. This holds promise as a way to further address the shortage of language-concordant providers. A recent 2017 report noted that only 36.2% of U.S. physicians speak Spanish, a proportion that has been replicated on the state level and has been cited as inadequate to meet the needs of the patient population [29, 30]. Educational curricula should be implemented to equip healthcare workers with skills necessary to appropriately care for such patients. Our findings suggest medical language courses are one potential way to modify the attitudes and enhance language skills of future physicians, encouraging them to care for patients with LEP. The impact of culturally sensitive and linguistically appropriate medical education on the attitudes of physicians throughout their careers needs further investigation.

Clinical electives abroad were rated favorably in terms of improvement in language proficiency and cultural understanding of the local in-country population, which is consistent with a recent finding that a Spanish immersion rotation improved fluency when compared to U.S.-based coursework alone [19]. Clinical immersion rotations are opportunities to consolidate one’s language skills in a clinical context, but require building and sustaining international partnerships. In a 2013 review of Medical Spanish programs, no consensus emerged regarding course delivery best practices. In addition, there was high variability in the frameworks used for the curriculum and assessment [31]. Given the overwhelmingly favorable ratings of clinical electives abroad and that such immersive rotations are superior to U.S.-based coursework alone, didactic U.S.-based course work (such as the month-long intensive Spanish course) combined with immersive clinical electives (whether abroad or local) should be further evaluated in medical language education. This approach is consistent with the literature regarding international medical graduates, which suggests that a combination of linguistic and clinical instruction can refine and enhance secondary medical language acquisition [32]. This underscores the need to compare different course modalities to determine which is most effective and practical.

Our study has several limitations. Our 26% response rate was low, but similar to other online survey response rates [33, 34]. The response rate may have been low because some of the email addresses were out-of-date. The short duration (4 weeks) over which the survey was available might have also contributed to this low response rate. However, the administration of the survey was discontinued at the end of 4 weeks because there was a sharp decline in the number of additional respondents after the second survey reminder email. Furthermore, physicians as a professional group are known to have low response rates to surveys [33]. When assessing respondents to non-respondents, we acknowledge that we were unable to compare graduation year, gender, or other demographic information between the groups. Additionally, our results may have been affected by social desirability and recall bias. Our respondents were skewed towards those who took a medical Spanish course, likely because of the higher prevalence of Spanish language speakers and more course options in Spanish. This resulted in a smaller sample size for the other courses, making it difficult to analyze differences in reported outcomes across different languages. However, because the framework of the longitudinal semester-long courses for all medical language courses is fundamentally the same, we were able to aggregate the data to assess outcomes across the Medical Language Program at HMS, rather than for individual language courses. The respondents were younger, with the majority less than 44 years of age (*n* = 152, 74%), which may reflect the increased availability of language courses after 2007. Nevertheless, a shorter time interval between course completion and survey administration could have resulted in higher proficiency ratings due to a lower likelihood of experiencing language attrition. Finally, while most of the survey items we used have not been administered in prior surveys, we developed the questionnaire through an iterative process with medical education evaluators and conducted pilot testing to maximize reliability of items.

## Conclusions

In summary, our study holds several implications for medical language education. Our findings suggest that medical schools should continue to offer or begin offering medical language courses that match the linguistic needs of the patient population. The courses may motivate students to continue working with linguistically diverse patients and may equip students to practice language-concordant care in their careers. Our data suggest that effective medical language courses may contribute to sustained learning and language use in professional settings over time. Our findings also suggest that providing formal assessments after course completions could be helpful in benchmarking progress toward clinical proficiency and professional competency. Finally, programs may consider offering clinical immersion electives through partnerships abroad as effective ways to help consolidate medical language learning, clinical training, and cultural understanding. These findings support continued development and investment in medical school language courses as a viable method to close gaps in physician-patient communication.

## Supplementary Information

**Additional file 1.** Survey.pdf: A pdf copy of the survey that was sent to eligible alumni.

**Additional file 2: Supplemental Table 1.** Most Commonly Expressed Themes in Responses to Open-ended Questions. Table summarizing the results of the qualitative coding of open-ended responses using the consolidated criteria for reporting qualitative research. The most commonly expressed themes in responses to open-ended questions are presented.

## Data Availability

Data used in analyses for this report are available by reasonable requests made to the corresponding author.

## Abbreviations

HMS: Harvard Medical School
LEP: Limited English proficiency
U.S: United States
