# Supplementary material for: Professional language use by alumni of the Harvard Medical School Medical Language Program
Source: BMC Med Educ. 2020 Nov 6;20:407. doi: 10.1186/s12909-020-02323-x (PMC7648424; doi:10.1186/s12909-020-02323-x)
Supplement: Supplementary file 1 — Additional file 1. Survey.pdf: A pdf copy of the survey that was sent to eligible alumni. [file 12909_2020_2323_MOESM1_ESM.pdf]

Career Trajectories of Alumni of the Medical Language Program (MLP) at Harvard Medical School

Introduction
We invite you to take this survey because of your past enrollment in one of the HMS Medical Language Program (MLP) courses, which include longitudinal semester-long language courses, intensive medical Spanish and/or clinical electives abroad (ME518), and Harvard Medical Language Initiative (HMLI) courses.

Your participation in this survey will help us improve the MLP courses for future students to deliver linguistically and culturally appropriate care. Your responses will be anonymous and the results will only be presented in aggregate. The survey should take between 5-10 minutes to complete.

To thank you for your time, we will give a $15 Amazon gift card to the first 150 respondents. We will ask your name and email address through a separate link at the end of the survey solely for the purpose of sending the $15 gift card. Your answers to the survey will not be associated with your name or email address. Thank you for your time. If you have any questions about the survey, please feel free to email Joe Pereira at Joseph_Pereira@hms.harvard.edu or Dr. Rose Molina at rmolina@bidmc.harvard.edu.

Instructions

If you took more than one language course at Harvard Medical School, please answer the questions in this survey as they pertain to the non-English language you have utilized most in your professional career. This language will be referred to as the “target language” in the survey. For example, if you took courses in Spanish and Mandarin, but you use Spanish more than Mandarin in your career, please answer the survey about the impact of the Spanish course(s) on your career.

Which of the following is the target language you are answering this survey about?

- ◯ Spanish
- ◯ Portuguese
- ◯ Mandarin
- ◯ Haitian Creole
- ◯ I did not take a language course at HMS

Thank you for your response, but unfortunately you are not eligible for the study. Please do not fill out the survey. Thanks for your time.

Of the following, which course(s) did you take in the target language while at HMS?

- ☐ A longitudinal (semester-long) medical language course
- ☐ An intensive (month-long) language course
- ☐ A clinical elective abroad
- ☐ Unsure
- ☐ Other

Please describe what "other" course you took:

____

What is your age?

- ◯ 25-34 years old
- ◯ 35-44 years old
- ◯ 45-54 years old
- ◯ 55-64 years old
- ◯ 65 years old and older

What is your gender identity?

- ◯ Female
- ◯ Male
- ◯ Self-described (other)

If you wish, please describe your gender identity: __________________________________

I identify with the following racial/ethnic groups:

- ☐ American Indian or Alaska Native
- ☐ Asian
- ☐ Black or African American
- ☐ Native Hawaiian or Other Pacific Islander
- ☐ White
- ☐ Hispanic, LatinX, or Spanish origin
- ☐ Other racial/ethnic group(s)

If you wish, please indicate the racial/ethnic group(s) you identify with: __________________________________

Your Linguistic Background

How would you describe your learning experience of the target language before taking the target language course at HMS? Check all that apply.

- ☐ Learned the language with family
- ☐ Studied the language in formal classes in school (e.g. high school, college)
- ☐ Studied the language in formal classes outside of school (e.g. after school programs, elective summer courses, online trainings)
- ☐ Lived/worked in a country where the target language was spoken
- ☐ Beginner student (e.g. only began learning the language several weeks or months before the start of the course)
- ☐ Other experience

Please indicate your other language learning experience(s):

____

How would you characterize your oral proficiency just before taking the language course(s) or clinical elective in the target language?

- ○ No practical speaking, listening, or reading proficiency
- ○ Elementary proficiency (basic courteous communication; read some names, signs, and numbers; understand isolated words and phrases)
- ○ Limited working proficiency (routine communication; read prose on subjects within a familiar context; speak about limited work-related topics)
- ○ Minimum professional proficiency (participate in most formal conversations with accurate grammar and vocabulary; read general newspapers; understand work-related correspondence, reports, and technical materials)
- ○ Full professional proficiency (fluent and accurate professional use, but the individual would not necessarily be perceived as culturally native)
- ○ Native or bilingual proficiency (equivalent to that of an educated native speaker)

How would you characterize your current oral proficiency in the target language?

- ○ No practical speaking, listening, or reading proficiency
- ○ Elementary proficiency (basic courteous communication; read some names, signs, and numbers; understand isolated words and phrases)
- ○ Limited working proficiency (routine communication; read prose on subjects within a familiar context; speak about limited work-related topics)
- ○ Minimum professional proficiency (participate in most formal conversations with accurate grammar and vocabulary; read general newspapers; understand work-related correspondence, reports, and technical materials)
- ○ Full professional proficiency (fluent and accurate professional use, but the individual would not necessarily be perceived as culturally native)
- ○ Native or bilingual proficiency (equivalent to that of an educated native speaker)

What are your primary or native languages? Check all that apply.	☐ English<br>☐ Spanish<br>☐ Portuguese<br>☐ Mandarin<br>☐ Other(s)
Please indicate your other primary or native language(s):	______________________________

Using non-English Languages in Your Current Professional Setting
Please answer the questions in this section as they pertain to the use of the target language in your current professional setting.

About how many hours per week do you spend speaking the target language?	◯ 0 hours per week<br>◯ >0 but < 1 hour per week<br>◯ 1-5 hours per week<br>◯ 6-10 hours per week<br>◯ 11-20 hours per week<br>◯ 21-40 hours per week<br>◯ 41+ hours per week
How many individuals do you speak to in the target language per week on average (including patients and colleagues)?	◯ 0 individuals per week<br>◯ 1-5 individuals per week<br>◯ 6-20 individuals per week<br>◯ 20+ individuals per week
With whom do you speak when using the target language? Please select all that apply.	☐ Patients<br>☐ Patients' family members<br>☐ Colleagues<br>☐ Other
Please specify the other(s) you speak to in the target language:	__________________________
Please describe your current occupation. Check all that apply.	☐ Clinician<br>☐ Research Investigator<br>☐ Medical Educator<br>☐ Other
As a clinician, please specify your current specialty:	__________________________
Please specify your occupation:	__________________________
Have you ever taken a formal language proficiency assessment for your workplace?	◯ Yes<br>◯ No
Was the assessment required for your work?	◯ Yes<br>◯ No
What was the name of the assessment (if known)?	__________________________

Participation in a Clinical Elective Abroad while at HMS
Please answer the questions in this section as they pertain to clinical electives abroad where the target language was predominantly used.

Did you participate in a clinical elective abroad while you were at HMS?	◯ Yes<br>◯ No
Did you use the target language during the clinical elective abroad?	◯ Yes<br>◯ No
How many weeks did you spend on that clinical elective?	◯ 1-3<br>◯ 4<br>◯ 5-7<br>◯ 8+<br>◯ Unsure
On a scale of 1 to 5, with 1 being "not at all" and 5 being "significantly improved," to what extent did the clinical elective improve your cultural understanding of the local population?	◯ 1 = Not at all<br>◯ 2<br>◯ 3<br>◯ 4<br>◯ 5 = Significantly improved
On a scale of 1 to 5, with 1 being "not at all" and 5 being "significantly improved," to what extent did the clinical elective improve your language proficiency of the target language?	◯ 1 = Not at all<br>◯ 2<br>◯ 3<br>◯ 4<br>◯ 5 = Significantly improved

The Impact of the Language Course on Your Career (Section 1 of 2)

Please answer the questions in this section as they pertain to the ways in which the target language course(s) and/or the clinical electives abroad where the target language was predominantly used may have impacted your career.

Before the course(s) began, how often did you expect to work with patients or other colleagues that spoke the target language in your career?

◯ Never
◯ Occasionally
◯ Frequently
◯ All the time

After the course(s) ended, how often did you expect to work with patients or other colleagues that spoke the target language in your career?

◯ Never
◯ Occasionally
◯ Frequently
◯ All the time

On a scale of 1 to 5, with 1 being "not at all helpful" and 5 being "significantly helpful," to what extent did the Medical Language Program course(s) help with your:

	Not at all helpful = 1	2	3	4	Significantly helpful = 5
Oral proficiency of the target language in your medical career	○	○	○	○	○
Oral proficiency of the target language in settings outside of medicine	○	○	○	○	○
Understanding the social determinants of health of those speaking the target language, including patients' social and cultural contexts	○	○	○	○	○

On a scale of 1 to 5, with 1 being "not at all motivated" and 5 being "significantly motivated," to what extent did each of the following motivate you to take the language course(s)?

	Not at all motivated = 1	2	3	4	Significantly motivated = 5
Desire to use the language as a part of future career	○	○	○	○	○
Desire to use the language in personal life apart from future career	○	○	○	○	○
Desire to learn more about social determinants of health of those who speak the language	○	○	○	○	○
Family/heritage influence	○	○	○	○	○

The Impact of the Language Course on Your Career (Section 2 of 2)
Please answer the questions in this section as they pertain to the impact of the language course(s) on your career. For each open-ended response, please write 2-3 sentences.

On a scale of 1 to 5, with 1 being "not at all influential" and 5 being "significantly influential," to what extent has the language course(s) at HMS influenced your career?	◯ 1 = Not at all influential<br>◯ 2<br>◯ 3<br>◯ 4<br>◯ 5 = Significantly influential
Please list two to three ways the course has influenced your career.	____
In what ways could the language courses be improved to better prepare medical students in delivering language-concordant care in their careers? Please list at least two suggestions.	____

Closing Questions

Please share your thoughts on the following questions.

How, if at all, did the language course help you better understand the culture of patients who speak the target language as it pertains to your current profession? __________________________________

Please share the estimated proportion of your patient population that speaks the target language (if applicable). __________________________________

Please share any other comments you may have about the HMS Medical Language Program. __________________________________
