# Supplementary material for: Professional language use by alumni of the Harvard Medical School Medical Language Program
Source: BMC Med Educ. 2020 Nov 6;20:407. doi: 10.1186/s12909-020-02323-x (PMC7648424; doi:10.1186/s12909-020-02323-x)
Supplement: Supplementary file 2 — Additional file 2: Supplemental Table 1. Most Commonly Expressed Themes in Responses to Open-ended Questions. Table summarizing the results of the qualitative coding of open-ended responses using the consolidated criteria for reporting qualitative research. The most commonly expressed themes in responses to open-ended questions are presented. [file 12909_2020_2323_MOESM2_ESM.docx]

Supplemental Table 1. Most Commonly Expressed Themes in Responses to Open-ended Questions

| **Influence of the medical language courses on professional career** |
| --- |
| Enabled and enhanced communication with patients and family members |
| Influenced choice of clinical setting or patient population |
| Allowed for appreciation and understanding of the culture of those who speak the target language |
| Increased comfort and confidence working with those speaking the target language |
| Helped build rapport and trust with patients |
| Improved general proficiency of language (including non-medical use) |
| **Ways the language courses could be improved** |
| Make the courses more intense, longer or longitudinal (additional resources and ongoing learning opportunities after courses end) |
| Offer more practical experience (Spanish Objective Structured Clinical Exam, exposure to Spanish-speaking patients, local shadowing opportunities, conversational practice with other students, individualized practice based on proficiency level) |
| Make the course more immersive (paired with clinical electives) |
| Include assessments (pre-course, during the course, and after the course) |
| **Value of language courses in facilitating understanding of the patient population** |
| Immersion courses (e.g. clinical electives abroad) were the best method of learning about socio-cultural contexts |
| Achieving language proficiency facilitated additional cultural learning after course ended (vocabulary and terminology from different countries and sensitivity to health literacy) |
| Socio-cultural issues were not taught at all, only minimally or ineffectively |
| Helped provide context for working with patient populations (understanding explanatory model of illness and verbal/social etiquette) |
